# Insulin-Induced Acanthosis Nigricans

**DOI:** 10.7759/cureus.83424

**Published:** 2025-05-03

**Authors:** Soomal Rafique, Hanan Dihowm, Sanober Parveen, Michael Jakoby

**Affiliations:** 1 Internal Medicine, Southern Illinois University School of Medicine, Springfield, USA; 2 Internal Medicine/Endocrinlogy, Southern Illinois University School of Medicine, Springfield, USA

**Keywords:** acanthosis nigricans, diabetes, hyperglycemia, insulin-induced, insulin-resistance

## Abstract

Acanthosis nigricans (AN) is a hyperkeratotic dermatosis characterized by hyperpigmented, velvety skin plaques, most commonly found on intertriginous sites such as the dorsal cervical region and axillae, though it can potentially occur on any skin surface. AN usually presents as a manifestation of systemic disorders associated with high-grade insulin resistance, such as obesity, metabolic syndrome, or type 2 diabetes mellitus (DM2). However, AN occurring at sites of subcutaneous insulin injections is rare.

We present a case of AN developing at sites of repetitive, high-dose insulin administration on the anterior abdomen. A 70-year-old male with DM2 was referred for evaluation of a “rash” at his insulin injection sites. He was prescribed a cumulative daily dose of 680 units of U-500 insulin and 80 units of insulin glargine U-300, all of which he reported injecting into the periumbilical area. Examination revealed a discrete, hyperpigmented, verrucous plaque surrounding the umbilicus, consistent with AN, with no other areas of affected skin. Histologic findings from a biopsy of the lesion showed papillomatosis and hyperkeratosis, features characteristic of AN. The patient was advised to avoid injecting insulin into the affected area and to rotate injection sites frequently. Modest improvement in AN was observed over the next three months, with more significant improvement during the subsequent six months following treatment with 0.1% retinoic acid cream.

## Introduction

Acanthosis nigricans (AN) is a hyperkeratotic dermatosis characterized by hyperpigmented, velvety plaques that may occur anywhere but most frequently on intertriginous skin of the dorsal cervical neck, axillae, below the breasts, and groin [1]. AN most commonly arises in conditions associated with significant insulin resistance, such as obesity, metabolic syndrome, polycystic ovary syndrome (PCOS), and type 2 diabetes mellitus (DM2). It is also observed in certain genetic syndromes characterized by severe insulin resistance, including Down syndrome, Rabson-Mendenhall syndrome, leprechaunism, and various forms of lipodystrophy [2,3]. There are multiple syndromes without insulin resistance in which AN may be observed including Crouzon syndrome, Costello syndrome, cutis gyrata syndrome, and thanatophoric dysplasia, and AN has been reported as a paraneoplastic manifestation of gastric adenocarcinoma [4,5]. Though rare, AN may occur at sites of subcutaneous insulin administration [6-16]. We present a case of AN occurring at sites of repetitive, high-dose insulin injections in subcutaneous skin of the anterior abdomen.

## Case presentation

**Figure 1:**
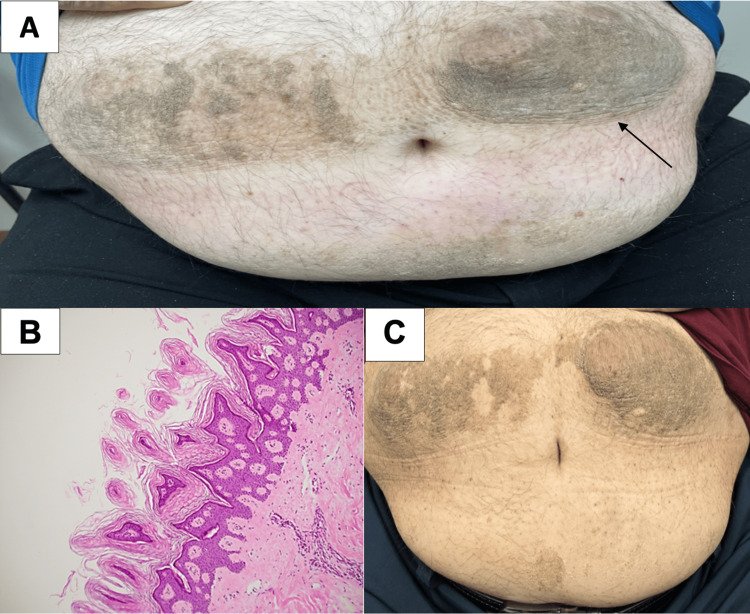
(A) Hyperpigmented, verrucous plaques on each side of the umbilicus with the appearance of acanthosis nigricans (AN), (B) histologic findings on biopsy of the affected area showing papillomatosis and hyperkeratosis characteristic of AN, and (C) improvement seen in AN after 12 months.

A 70-year-old male with a 20-year history of DM2 was referred for management of poorly controlled hyperglycemia. Hemoglobin A1c (HbA1c) at time of referral was 9.8%, and the patient reported full compliance with a regimen of U500 regular insulin (U500) 680 total units daily, 80 units of insulin glargine U300 at bedtime, and liraglutide 1.8 mg weekly. On examination, the patient was observed to have an obese abdomen with two discrete hyperpigmented, verrucous plaques on each side of the umbilicus with the appearance of AN (Figure 1A). 

No other plaques were observed in areas frequently affected by AN (e.g., dorsal cervical neck and axillae). The patient reported that he had been administering insulin exclusively into periumbilical skin for many years. 

Insulin-induced AN was suspected, and the patient was referred to the dermatology clinic for further evaluation. Shave biopsy of a plaque demonstrated histologic findings showing papillomatosis and hyperkeratosis characteristic of AN (Figure 1B) [4]. 

Administration of insulin remote from sites of AN and rotation of insulin administration sites was recommended, and modest improvement in AN was noted on examination three months later. More significant improvement occurred at 12 months from diagnosis of AN after treatment with 0.1% retinoic acid cream, though plaques still remained fairly extensive (Figure 1C). Insulin dose requirements decreased significantly after the patient moved sites of insulin administration away from the umbilicus, with insulin aspart substituted for U500 insulin to provide prandial insulin coverage and insulin glargine U300 continued for basal insulin coverage (Table 1). 

**Table 1:** Insulin requirements and glycemic control after diagnosis of insulin injection site acanthosis nigricans (AN) HbA1C: Hemoglobin A1c

Time	Total daily insulin dose (units)	HbA1c (%)	Normal Value (%)
Diagnosis of AN	790	9.8	< 5.6
3 months after diagnosis	250	9.7	< 5.6
12 months after diagnosis	32	8.5	< 5.6
15 months after diagnosis	70	7.9	< 5.6

Dulaglutide 1.5 mg weekly was substituted for liraglutide, and empagliflozin 25 mg daily was added to the patient’s glycemic regimen. Glycemic control was measurably improved 12 months after changes to sites of insulin administration and therapeutic agents were made (Table 1).

## Discussion

The underlying mechanisms of AN are not fully understood, but it is believed that in conditions marked by severe insulin resistance, such as obesity, elevated insulin levels may activate IGF-1 receptors. This activation promotes the proliferation of keratinocytes and fibroblasts, resulting in the characteristic skin changes seen in AN [4]. In some series, AN is present in 35% of DM2 patients [7]. Drug-induced AN is an uncommon subtype. A systematic review of 38 studies identified 13 drugs associated with the induction of acanthosis nigricans, with nicotinic acid and insulin emerging as the most prominent contributors [6]. Nevertheless, AN at insulin injection sites is rare and exclusively documented in case reports [8-16].

In cases of insulin injection-induced AN, repeated exposure of subcutaneous tissue to high concentrations of insulin can lead to the development of AN [8]. Despite obesity, this patient had AN only at sites of abdominal wall insulin administration, implicating exogenous insulin as the etiology of AN.

The most important clinical impact of anterior abdominal wall AN was inability to control hyperglycemia despite treatment with high doses of U500 insulin, presumably due to impaired insulin absorption at sites of insulin administration from skin changes of AN [8-9,16]. Total daily insulin dose was reduced by more than twofold (~450 units daily) after the patient began administering insulin remote from the peri-umbilical region affected by AN, providing an approximation of the extent to which AN at sites of insulin administration adversely affected the patient’s daily insulin requirement. The fact that total daily insulin dose remained high (200-300 units daily) after changing insulin injection sites reflects the systemic insulin resistance of DM2 and obesity (patient’s BMI 44 kg/m2). Though dulaglutide, a GLP-1 receptor agonist, and empagliflozin, an SGLT2 inhibitor, are useful therapeutic adjuncts for patients with DM2 who require insulin for management of hyperglycemia, neither agent individually nor the two drugs in combination accounts for the patient’s large reduction in daily insulin requirement. The patient was already managed with another GLP-1 receptor agonist (liraglutide) at start of treatment when insulin dose requirement was highest, and an exploratory analysis of EMPA-REG OUTCOME trial data showed that only a small proportion (9.2%) of patients receiving empagliflozin achieved a ≥ 20% reduction in total daily insulin dose without a decrement in glycemic control [17]. 

Improvement in abdominal wall AN for this patient was modest to moderate despite cessation of insulin administration at sites of affected skin and treatment with a topical kerolytic agent. Case reports of AN at insulin injection sites report both partial and complete resolution of AN after cessation of insulin dosing in affected skin, including one patient who experienced resolution of AN eight months after using alternative sites for insulin administration followed by recurrence of AN two months following resumption of serial same-site insulin dosing [15].

## Conclusions

This patient’s case illustrates that repetitive administration of insulin in a localized area can result in injection-site AN. Occurrence of AN may interfere with insulin absorption and increase total daily insulin requirement if patients continue to dose insulin in affected skin. Administration of insulin at sites remote from areas of AN significantly improves total daily insulin requirement, presumably due to more timely and complete absorption of exogenous insulin doses. Cessation of insulin dosing at sites of AN improves skin changes, though AN may not completely resolve.
